# The Link Between Personal Values and Frequency of Drinking Depends on Cultural Values: A Cross-Level Interaction Approach

**DOI:** 10.3389/fpsyg.2018.01379

**Published:** 2018-08-07

**Authors:** Maksim Rudnev, Christin-Melanie Vauclair

**Affiliations:** ^1^National Research University Higher School of Economics, Moscow, Russia; ^2^Instituto Universitário de Lisboa (ISCTE-IUL), CIS-IUL, Lisbon, Portugal

**Keywords:** basic human values, cultural values, drinking, alcohol, European Social Survey

## Abstract

The increasing availability of large cross-national datasets enables researchers to integrate micro and macro levels of relations between human values and behavior. Particularly interesting are interactions between personal and cultural levels which can demonstrate to what extent a specific behavior is affected by individual values and cultural context. In this study, we aimed to shed light on this issue by analyzing data on basic values and drinking behavior from 21 national representative samples of the European Social Survey (2014). The results of multilevel regressions showed that country-level effects of Openness to Change (vs. Conservation) or Self-Transcendence (vs. Self-Enhancement) were not significantly related to frequency of drinking. As expected, individual-level Openness to Change (vs. Conservation) was consistently positively related to drinking frequency, whereas Self-Transcendence (vs. Self-Enhancement) was not. Contrary to our hypothesis, there was a positive association between personal Self-Transcendence (vs. Self-Enhancement) values and frequency of drinking in countries putting higher importance on extrinsic motivations (i.e., Conservation or Self-Enhancement values), while this link was less positive or even negative in countries valuing intrinsic motivations (i.e., Openness to Change or Self-Transcendence values). Moreover, a marginally significant interaction between individual- and country-level Openness to Change (vs. Conservation) values supported the same counter-intuitive result. These findings challenge the widespread idea that more conservative societies attenuate the link between personal values and behavior. In contrast, self-affirmation and cultural rewards theories, as well as culture-specific value instantiations, may explain these results. This study shows that the value-behavior link differs across cultures, yet in a more complex way than was assumed so far. This opens up new possibilities for research on values and behavior in a cross-cultural context.

## Introduction

The increasing availability of large cross-national datasets enables psychologists to integrate micro and macro levels of analysis. Particularly interesting are cross-level interaction effects which show whether the nature of a lower-level relationship depends on a higher-level variable. These kinds of cross-level interactions can illustrate to what extent the link between values and behavior changes as a function of context characteristics. Although personal values have been found to be associated with a large variety of behaviors, little is known regarding how culture shapes these relationships (Roccas and Sagiv, 2010). To date, there have been only a few studies that examined cross-level interaction effects for value-behavior relations (e.g., Vecchione et al., 2015). To the best of our knowledge, no study has investigated how the interaction of country-level and individual-level values is associated with behavior. In this study, we aim to shed light on this issue by examining the interplay of personal and cultural values in predicting drinking behavior.

Intensity (or quantity) of drinking is one of the dimensions of alcohol consumption. It is usually addressed in health research because of its consequences for mental and physical health. Another crucial dimension of alcohol consumption is the frequency of drinking, which is interesting from a social-psychological perspective, because it can serve as an expression of people's motivational goals. Furthermore, this relationship may be moderated by the cultural context, so studying it can provide further insight into the value-behavior link as a function of culture.

To date, researchers have focused on socio-demographic and psychological factors, such as age and personality traits, as predictors of drinking behavior. The social context in which drinking behaviors are situated has been mostly examined at the meso-level, e.g., in the form of neighborhood characteristics that foster frequent drinking behaviors (Sudhinaraset et al., 2016). With a few exceptions (e.g., Inman et al., 2017), societal-level factors have been mainly studied in the form of policies related to alcohol use or in terms of alcohol advertisement and marketing. Consequently, there is very little research about how cultural factors relate to alcohol consumption. Most importantly, to date no study has examined how the link between personal values and alcohol consumption may depend on the cultural context. The present paper aims to fill this gap by investigating the association of personal values with alcohol consumption across European countries and examining how this value-behavior relation is moderated by cultural values.

Studies on alcohol consumption can contain a wide variety of different items to assess self-reported drinking behavior, such as drinking status (drinking vs. not drinking), intensity or quantity of drinking (e.g., average volume of alcohol consumption over a given period of time) and frequency of drinking, as well as quantity and frequency of heavy or binge drinking. These can be summarized by two overall dimensions: frequency of drinking and intensity of drinking or extent of drunkenness (Room and Mäkelä, 2000). Past research has shown that frequency and intensity of drinking show different relations with key socio-demographic variables: although it is a universal trend that men consume more alcohol and do so more frequently than women (Mäkelä et al., 2006), younger people tend to drink less frequently but in greater quantity than older people (WHO, 2014); people with higher socio-economic status (SES) tend to drink more frequently, but in smaller quantities than low-SES groups (Sudhinaraset et al., 2016).

In this study, we focus on the frequency and not the intensity of drinking alcohol. We are interested in examining variation in the use of a recreational lifestyle substance as it applies to most people in society, without focusing on the extent of drunkenness, which is theoretically less related to personal and cultural values and more related to health and addiction issues. Frequency of drinking reflects a more conscious behavior that in turn may be based on people's motivational goals. Moreover, the frequency of drinking is a much more reliable self-reported measure of behavior than the amount of drinking, and reliability is a crucial issue when examining value-behavior relations. Questions about the amount of drinking are highly sensitive and usually underestimate the real amounts (for a review see Nugawela et al., 2015). Previous analyses of European Social Survey (ESS) data have also shown that questions about quantity of drinking and abstinence were much more subject to interviewer bias than questions about frequency of drinking (Wuyts et al., 2016).

Why do people drink alcohol? When it comes to psychological predictors of alcohol consumption, drinking behavior has been widely explained in terms of immediate self-reported drinking motives. Researchers generally agree about the following motives for alcohol consumption: (1) enjoyment and sensation-seeking, (2) social motives, and (3) coping motives, related to anxiety and coping with stress (Abbey et al., 1993; Kuntsche et al., 2006; Mobach and Macaskill, 2011). Some researchers also distinguish a so-called conformity motive. Grant et al. (2007) found that enjoyment and social motives demonstrated stable positive associations with drinking frequency, whereas conformity motives showed negative and coping showed non-significant associations. Cross-national studies on drinking motives also found that social and enjoyment motives were generally positively related to frequency of drinking, while conformity motives were negatively related to it (Kuntsche et al., 2014).

In sum, the literature on drinking motives shows that motives are strong predictors of drinking behaviors, even more so than socio-demographic variables (Grant et al., 2007).

Different motivational goals have been suggested to be at the core of different types of values which drive attitudes and behavior (Schwartz, 1992). Hence, it is conceivable that there is also a link between values and alcohol consumption.

Although there are numerous value theories in the literature (e.g., Rokeach, 1973; Hofstede, 2003), the one that claims to cover most universal values, elaborated and tested across many cultures was proposed by Schwartz (1992, 2007). This theory assumes value conflicts and compatibilities at both the personal and societal level. Its alignment with motivational goals allows for development of specific hypotheses at different levels about the association between values and behavior. Schwartz (1992) defines values as abstract desirable goals that serve as guiding principles in people's lives. He argues that basic values can be organized into two higher-order dimensions and each dimension is marked by a bipolar value orientation which represents the fundamental value conflict. One dimension is labeled Openness to Change (vs. Conservation) and relates to the conflict of being motivated either to pursue autonomous, self-expressive experiences (intellectual and emotional) or to emphasize order, self-restriction, preserving the status quo (Schwartz, 1992). The second dimension is labeled Self-Transcendence (vs. Self-Enhancement) and represents the conflict of being motivated either to transcend selfish concerns and promote the welfare of others (both close and distant, as well as the environment) or to enhance one's own personal interests (even at the expense of others). Schwartz (1992) proposes ten specific values that are organized in line with these two higher-order value dimensions: Self-Direction, Stimulation and Hedonism (for Openness to Change); Conformity, Security and Tradition (for Conservation); Power and Achievement (for Self-Enhancement); and Benevolence and Universalism (for Self-Transcendence).

Schwartz (1994, 2007) also develops a culture-level value theory consisting of three fundamental value conflicts: Autonomy vs. Embeddedness, Harmony vs. Mastery, and Egalitarianism vs. Hierarchy. He acknowledges that these culture-level values parallel individual-level values, such as Universalism being similar to Egalitarianism, or Conservation values approximately matching Embeddedness (Schwartz, 2007), and that it is reasonable to assume that there is some structural isomorphism between values at the individual and the culture levels (Schwartz, 1994). In fact, several studies have shown that the individual-level value types can be aggregated and meaningfully used at the culture level without substantial loss of information (Fischer et al., 2010; Fischer and Poortinga, 2012; Fischer, 2013). Using the same value types at both levels of analyses in multilevel models allows researchers to ask new questions about the role of personal and cultural values in shaping people's behavior: are the same value types associated in the same ways with the behavior across levels of analysis or do they show different patterns of association? Do the same value types at the individual and culture levels interact with each other to exacerbate or mitigate their effect on individuals' behavior? Such research questions cannot be investigated if value types are conceptualized as being inequivalent across levels of analysis. Hence, in this study we examine the same value types as predictors of drinking behavior at the individual and culture levels.

A few studies have already examined the link between personal values and various characteristics of drinking. A pioneering study by Gorsuch and Arno (1979) suggested that values endorsing group interests were negatively related to problematic alcohol consumption and casual drinking, whereas tolerance toward deviance had a positive association with positive attitudes to drinking. Since then, only a few studies have been conducted on the link between values and drinking, as shown in our systematic literature search in Google Scholar, PubMed, and Web of Science databases, using “basic values,” “alcohol,” and “drinking” keywords. For the sake of better comparability, we focused on studies that used Schwartz basic values or ones easily coerced to Schwartz theory. After excluding duplicated reports, we identified 25 studies. With the aid of the reference lists in these papers, we found additional three studies. Finally, we removed studies that did not include indicators of alcohol consumption or attitudes toward it, resulting in 10 papers which are summarized in Table 1.

**Table 1:** Summary of previous studies of association between basic values and indicators of alcohol consumption at the individual level.

**Study**	**Conservation**			**Self-Transcendence**		**Openness to Change**			**Self-Enhancement**		**Type of sample**	**Measure of alcohol consumption**	**Values instrument**
	**SE**	**CO**	**TR**	**BE**	**UN**	**SD**	**ST**	**HE**	**AC**	**PO**			
Kropp et al., 1999	–	*ns*	*ns*	*ns*	*ns*	*ns*	+	*ns*	*ns*	*ns*	257 students from USA, Canada, and Australia	The fact of beer drinking	^*^ Non-Schwartz List of Values scale
Schwartz et al., 2001	–	–	–	*ns*	*ns*	+	+	+	*ns*	*ns*	South African national sample, 3210	Number of brands of alcoholic beverages ever used	PVQ-29
Dollinger and Kobayashi, 2003	–	–	–	–	–	+	+	+	*ns*	+	156 US midwestern university students	Ever had a drinking binge + ever drove drunk	SVS-56
Kropp et al., 2004	–	*ns*	*ns*	*ns*	*ns*	*ns*	+	*ns*	–	–	692 university students from South Korea, Canada, Australia, and USA	Positive attitude toward drinking	^*^Non-Schwartz List of Values scale
Cole et al., 2007	–	*ns*	*ns*	*ns*	–	*ns*	*Ns*	*ns*	*ns*	+	689 Bahamian sixth-grade students	If ever tried alcohol	PVQ-39
Ramírez and Musitu, 2008	*ns*	–	–	*ns*	*ns*	*ns*	+	+	*ns*	*ns*	350 Mexican adolescents	AUDIT scale combining frequency, quantity and problem drinking (WHO, 2001)	SVS-57
Lam, 2010	–^*^	–^*^	–^*^	–^*^	–^*^	+^*^	+^*^	*ns*	ns^*^	ns^*^	1,385 secondary school students in Hong Kong	Frequency of drinking	PVQ-40
Sheppard, 2011, p. 104	–	–	–	–	–	*ns*	+	+	+	+	910 US southeastern students	Attitudes toward drinking	PVQ-40
Anderson, 2012	*NA*	–	–	*ns*	*ns*	*NA*	*ns*	+	*NA*	*ns*	131 US college students	Absence of intention to drink moderately	PVQ-40
Nordfjærn and Brunborg, 2015 (Table 6)	*ns*	–	–	*ns*	–	*ns*	*ns*	+	+	*ns*	3,179 Norwegians aged 40 to 79	Combined frequency and quantity of consumption	PVQ-21
Share of ns	3/9	3/10	3/10	7/10	5/10	6/9	3/10	4/10	6/9	6/10			

*The coefficients themselves are not reported because they come from different statistical procedures, and therefore are not comparable*.

* *The higher-order values were used, i.e., Openness to Change, Self-Transcendence, etc. ns, Not significant. NA, was not assessed. SE, security; CO, conformity; TR, tradition; BE, benevolence; UN, universalism; SD, self-direction; ST, stimulation; HE, hedonism; AC, achievement; PO, power*.

These studies are very diverse in their design, participants, measures of drinking and values. However, there is a consistent pattern where Openness to Change values (Hedonism, Stimulation, and to some extent Self-Direction) are positively related to alcohol consumption, and Conservation values (Security, Conformity, Tradition) are negatively related. This is the case for assessments of drinking status used with adolescents in Mexico and Hong Kong (drinking vs. not drinking; Ramírez and Musitu, 2008; Lam, 2010) and of the number of alcoholic drink brands consumed among South Africans (Schwartz et al., 2001). In a similar vein, Sheppard (2011) showed that positive attitudes toward drinking were positively related to Stimulation, Hedonism, Achievement, and Power values, and negatively to Tradition, Conformity, and Universalism, and somewhat less negatively to Security and Benevolence. Anderson (2012) found that US students who self-monitored their alcohol consumption (i.e., limited their drinking) were less likely to value Hedonism and endorsed Conformity and Tradition. Studies examining frequency of drinking among adolescents or young adults also found that endorsing Conservation values was negatively related to frequent alcohol drinking (Young and West, 2010). This trend holds even if middle-aged and older adults are surveyed, as was done in a study by Nordfjærn and Brunborg (2015) with Norwegian respondents. Studies that did not use the Schwartz survey to assess values, but related value constructs, also reported a negative link between drinking and tradition-related values among respondents from Croatia, Hungary, and the USA (Unger et al., 2002; Pikó and Brassai, 2007; Livazović and Jukić, 2017).

The findings on values reflecting the higher-order value dimension Self-Transcendence (vs. Self-Enhancement) showed less consistent or non-significant associations with drinking behavior. Some studies showed that Self-Transcendence values were associated with abstinence (although weakly in Lam, 2010) and were not endorsed by frequent and problematic drinkers (Dollinger and Kobayashi, 2003). Regarding the Self-Enhancement values, it appears that Power values were positively related to various drinking behaviors (Dollinger and Kobayashi, 2003; Cole et al., 2007; Sheppard, 2011), except in Nordfjærn and Brunborg (2015) study of older adults. Achievement values were positively related to alcohol consumption in some studies (Sheppard, 2011; Nordfjærn and Brunborg, 2015), but not in others (Dollinger and Kobayashi, 2003). Studies with related value constructs (but not in the framework of Schwartz value theory) also reported some evidence between drinking and power-related values (namely sexist authoritarianism and material values; Unger et al., 2002; see Livazović and Jukić, 2017).

Overall, it seems that Conformity and Tradition (belonging to the Conservation higher-order value) are robust negative correlates of alcohol consumption, whereas Hedonism and Stimulation (belonging to the Openness to Change higher-order value) are strong positive correlates. This is consistent with the findings related to the enjoyment drinking motive (see above), as enjoyment-seeking is a direct expression of Hedonism and Stimulation values. It is also reasonable from a psychological perspective: drinking is universally associated with enjoyment-seeking and approach motivations, but not compatible with values emphasizing self-control as well as avoidance and prevention of harm (Ostafin et al., 2003). Hence, we expect that:

H1: The value dimension Openness to Change (vs. Conservation) is positively related to the frequency of drinking at the individual level.

Based on differences between basic values in their relation to either approach or avoidance motivations, it would be reasonable to expect that Self-Transcendence values are positively related to the frequency of drinking, while Self-Enhancement values are negatively related. However, this does not correspond to the findings of the above studies, which show the opposite associations or none at all. Therefore, regarding the Self-Transcendence (vs. Self-Enhancement) value dimension, we did not hypothesize its relation to frequency of drinking at the individual level but merely explored the association.

Studies examining the cultural context of alcohol use mainly focus on differences between ethnic groups which are explained *post-hoc* with differences in cultural beliefs and norms (see Sudhinaraset et al., 2016). There are only a few studies that specifically examine cultural values and their associations with alcohol consumption. A study across 42 countries revealed that countries characterized as being individualistic-oriented according to Hofstede's value dimensions also had higher rates of beer consumption than countries characterized as collectivistic (Zhang and Shrum, 2009). Mackinnon et al. (2017) found that students from more individualistic countries rated all drinking motives more positively than those from collectivistic countries, reflecting the emphasis on approach motivations to drinking in individualistic societies. In a similar vein, Inman et al. (2017) demonstrated that there was a significant link between per capita alcohol consumption across 74 countries and Schwartz cultural values. They found that Affective and Intellectual Autonomy, as well as Harmony values, were positively related and Embeddedness and Hierarchy values were negatively related to per capita alcohol consumption. Intellectual Autonomy and Egalitarianism showed positive, but marginally significant associations with alcohol consumption. Interestingly, in the subsample of European countries, only Embeddedness and Harmony showed significant relations with alcohol consumption, although the directions and magnitudes of associations were similar to the ones in the general sample. However, findings are still somewhat inconclusive; for example, Mackenbach (2014) did not find significant associations between cultural values and overall alcohol consumption in European countries, although the direction of effects suggests that people in countries with high Embeddedness and Egalitarianism drink less alcohol. Overall, the findings suggest that European countries emphasizing conservative values (such as the Embeddedness cultural value) are likely to show lower overall frequency of drinking than countries emphasizing Openness to Change values (such as the Affective and Intellectual Autonomy cultural values). Based on Inman's et al. results 2017, it is also reasonable to expect that individuals in countries emphasizing Self-Transcendence values (such as the Harmony and Egalitarianism cultural values) and de-emphasizing Self-Enhancement values (such as the Hierarchy cultural value) report somewhat more frequent drinking. Therefore, we hypothesize that:

H2: The value dimension Openness to Change (vs. Conservation) is positively related to the frequency of drinking at the country level.
H3: The value dimension Self-Transcendence (vs. Self-Enhancement) is positively related to the frequency of drinking at the country level.

In the studies reviewed above, cultural values were examined in terms of having a direct effect on alcohol consumption. However, cultural values can also exert a cross-level interaction effect by changing the strength of the relationship between two individual-level variables. There is some indication that individual-level associations with drinking behaviors may not be the same across different cultures. Kuntsche et al. (2014) reveal that the motive-drinking link differs between students aged 17–19 from different parts of Europe: social motives show a stronger link with frequency of drinking among Northern European students, while the association with enjoyment and conformity is stronger in Southern Europe. However, among younger students, these differences are reversed. Taken together, the empirical evidence on the variability of these individual-level associations is scarce, inconclusive and has focused on drinking motives rather than values.

From a theoretical standpoint, culture constitutes a powerful normative context that can provide a greater or lesser sense of legitimacy for acting in line with one's personal values. Moreover, a culture can directly reward or discourage specific behaviors. In other words, culture may moderate the value-behavior link by enhancing or attenuating it (Bardi and Schwartz, 2003). Personal values are individuals' internal compasses that guide them in their selection of possible behaviors (Schwartz, 1996). In cultures that value intrinsic motivations, internal attributes in the form of personal values are likely to affect behavior more strongly. However, the guidance function of personal values may be compromised in cultures in which intrinsic motivations, and therefore acting upon one's personal values, are less important (Roccas and Sagiv, 2010). Cultures that are characterized by stricter societal norms, such as collectivistic or “tight” societies, regulate individuals' behavior more strictly (Gelfand et al., 2011). In these cultures, individuals are more likely to take social norms and expectations into account when it comes to their behavioral choices, and so the value-behavior link becomes attenuated (cf. Vauclair and Fischer, 2011). In contrast, in more “loose” societies, the culture's regulation of behaviors is less pronounced. In combination with a norm of higher autonomy, this leads these societies to encourage individuals to express their own values.

Values at the individual level can also interact in affecting behavior. For example, Lönnqvist et al. (2006) show that Conformity values weaken relations between Self-Transcendence and altruistic behavior. Individuals low in Conformity are more reluctant to conform to social norms, so they feel free to express their Self-Transcendence values. Given that individual-level Conformity is affected by culture-level Conformity, it is reasonable to expect culture-level Conformity values to have a moderating effect on the link between personal values and behavior.

Schwartz's (2015) value theory also draws a distinction between intrinsic and extrinsic motivation which is crucial to understanding how cultural values might moderate the value-behavior link. Openness to Change and Self-Transcendence values are characterized by intrinsic motivation. Behaviors that express these values provide satisfaction or pleasure through expressing autonomy and competence (Openness to Change) or nurturance and relatedness (Self-Transcendence). On the other hand, Conservation and Self-Enhancement values are characterized by extrinsic motivation, and attainment of these values is regulated more by obtaining social approval and material rewards (Self-Enhancement), meeting the expectations of others and avoiding the sanctions they may impose, or receiving protection and care (Conservation). Therefore, cultures that promote intrinsic motivations by emphasizing Openness to Change and Self-Transcendence values encourage people to express their values. In contrast, cultures that promote extrinsic motivations by emphasizing Conservation and Self-Enhancement values prevent people from expressing their values in general, encouraging them to follow normatively prescribed behaviors regardless of their values. Assuming that frequent drinking is a socially undesirable behavior in conservative cultures (see Inman et al., 2017), we would expect that the link between values and frequency of drinking is stronger in cultures that emphasize intrinsic motivations than in those that emphasize extrinsic ones. Hence, we hypothesized that:

H4: The individual-level value dimension Openness to Change (vs. Conservation) has a stronger positive relation to frequency of drinking in cultures characterized by higher Openness to Change (vs. Conservation).

Given the inconsistency of individual-level findings in regard to the Self-Transcendence (vs. Self-Enhancement) dimension, we did not formulate any hypothesis. However, following the extrinsic-intrinsic rationale presented above, we expected that value-behavior links may be stronger in cultures valuing Self-Transcendence than Self-Enhancement.

## Data and method

We analyzed data from the seventh round of the ESS (Jowell et al., 2007) which was collected in 2014 in 21 European countries. Each country was surveyed with a representative national sample of persons aged 15 and above. The total number of respondents in the analysis is 37,121. The countries, sample sizes, average age, and gender balance by country are listed in Supplementary Materials, Table S1.

An ESS module team developed a questionnaire on alcohol consumption (Eikemo et al., 2017) assessing the frequency of drinking with the following question: “In the last 12 months, that is since [MM, YY], how often have you had a drink containing alcohol? This could be wine, beer, cider, spirits, or other drinks containing alcohol”. The response option included a 7-point scale from “Every day” to “Never” (see Figure S1 in Supplementary Materials for a full list of options). The scale was recoded so that higher scores reflected higher frequency of drinking. For ease of interpretation, the variable was standardized across the whole sample so that the overall mean was zero and 1 unit stood for one standard deviation.

Values were measured with the Portrait Values Questionnaire (Schwartz et al., 2001) as assessed in the ESS. The values were measured with 21 value portraits, each of which was evaluated by respondents on a six-point similarity scale with the following options: “Not at all like me,” “Not like me,” “A little like me,” “Like me,” and “Very much like me.” The scale was recoded so higher scores reflected a higher importance of the corresponding value. At the individual level, we used two higher-order value dimensions: Openness to Change vs. Conservation and Self-Transcendence vs. Self-Enhancement. We made this choice because some higher-order values showed partial cross-country measurement invariance (Davidov et al., 2008a; Cieciuch et al., 2016), and pairs of these higher-order values showed consistent conflicts between Conservation and Openness to Change, and between Self-Enhancement and Self-Transcendence (Fontaine et al., 2008; Rudnev et al., 2018). The value dimensions were computed as a difference score between the Openness and Conservation mean index, as well as the Self-Transcendence and Self-Enhancement mean index. In order to test the robustness of the main results, the analyses were replicated with the four higher-order values. In addition, we repeated the analysis with the ten value indices, although their cross-cultural comparability (measurement invariance across countries) is questionable (see Davidov et al., 2008b). We treated these results with caution and drew on them only as an additional check for our main conclusions (see Tables S3–S4). At the country level, we used country averages of the individual-level value dimensions. This was justified as they had shown cross-level isomorphism in previous research (Fischer, 2012). In order to separate individual and country-level variances of values, we applied different centering methods between the two levels of analysis. The individual-level values were group-mean centered, whereas country-level values were grand-mean centered and divided by their standard deviations.

We also included several control variables that are commonly known to be related to both frequency of drinking and basic values. These are gender, age, education (e.g., Huijts et al., 2017), relationship status (Nordfjærn and Brunborg, 2015), frequency of social encounters (as evidenced by research on social motives, e.g., Kuntsche et al., 2014), religiosity (Cochran, 1992) and depression (e.g., Holahan et al., 2003). All individual-level covariates (except for depression and dummy coded variables) were grand-mean centered so that zeros stand for the population mean, which in turn provides more correct estimates of random intercepts and facilitates their interpretation.

Education was measured with overall years of schooling standardized across the whole sample so that between-country variance was left in the data. Relationship status was a dichotomous variable indicating whether respondent lived with a partner. Frequency of social encounters was measured with a single question on the frequency of intentional social meetings with friends, relatives, or colleagues on a 7-point scale from “Never” to “Every day.” Religiosity was measured by a question about how religious a respondent was on the scale from “Not at all religious (0)” to “Very religious (10).” Depression was measured with an eight-item version of the Center for Epidemiologic Studies Depression Scale (Radloff, 1977). The items captured frequency of symptoms such as “Your sleep was restless” or “You were happy,” with 4 response options each from “None or almost none of the time” to “All or almost all of the time.” We conducted a multiple group confirmatory analysis in order to test measurement invariance of the scores and found partial metric measurement invariance (allowing the factor loading of the lack of self-motivation item (“could not get going”) to vary across countries; see Table S2). This level of measurement invariance is sufficient to use the factor score as a predictor variable in a regression. The scale is reversed so that higher scores reflect higher happiness and lower depression. Due to the metric invariance analyses, factor means were constrained to 0 in every group, so the obtained index (factor scores) had a mean of zero in every country and therefore did not have to be grand-mean centered.

### Analytical strategy

We employed multilevel regressions because the data is clustered within countries (Hox, 2010). The clustering violates the assumption of independent observations for ordinary linear regression. The number of countries in the sample (21) is below that required for reliable estimates of country-level effects and cross-level interactions with usual maximum likelihood estimation (Stegmueller, 2013), so we used bootstrapping to find standard errors of the estimates, and conducted analysis with a Bayesian estimator as a robustness check. The data was weighted with design weights to correct for the differences in the probability of different members of the population of being included in the sample. All the analyses were conducted in R, mostly using packages “lme4” (Bates et al., 2018) and “brms” (Bürkner, 2017).

## Results

The overall frequency of alcohol consumption varies substantially across countries (see Figure S1).

Examination of correlations between value dimensions and frequency of drinking (see Table 2) showed that, in line with the literature reviewed above and our hypotheses, Openness to Change (vs. Conservation) values were positively related to drinking at both individual and country levels. Self-Transcendence (vs. Self-Enhancement) values were also positively related to frequency of drinking at the country level, while at the individual level in most countries in our sample the correlations were negative. There were countries with a positive link between Self-Transcendence (vs. Self-Enhancement) and frequency of drinking, such as Belgium and France. This suggests that there is a fair amount of cross-country variability in these relationships.

**Table 2:** Correlations at the individual and country levels between frequency of drinking and basic values.

	**Openness to Change (vs. Conservation)**	**Self-Transcendence (vs. Self-Enhancement)**
Average within-country (standard deviation)	0.15 (0.07)	−0.04 (0.05)
Country-level	0.62^**^	0.65^**^

** *significant at p < 0.01*.

The multilevel model was built in several steps. First, we fitted an empty model and found that 8% of the variance of frequency of drinking was due to cross-country differences (as indicated by the intraclass correlation). At the second stage, we entered all the control variables and tested their effects for differences across countries. Effects of gender and age were found to differ substantially across countries, so we included their random effects in all the subsequent models. At the third stage, we entered country-level values, random effects of individual-level values, and finally, interactions between individual- and country-level values. The results are listed in Table 3.

**Table 3:** Multilevel regression coeffients, dependent variable: frequency of drinking.

	**(M1)**	**(M2)**	**(M3)**	**(M4)**	**(M5)**	**(M6)**	**(M7)**
**INDIVIDUAL LEVEL**							
Openness to Change (vs. Conservation)	0.10^***^ (0.005)	0.10^***^ (0.005)	0.10^***^ (0.01)	0.10^***^ (0.01)	0.10^***^ (0.01)	0.10^***^ (0.01)	0.10^***^ (0.01)
Self-Transcendence (vs. Self-Enhancement)	0.002 (0.005)	0.001 (0.005)	0.004 (0.01)	0.004 (0.01)	0.004 (0.01)	0.005 (0.01)	0.004 (0.01)
Female	−0.45^***^ (0.04)	−0.45^***^ (0.04)	−0.45^***^ (0.04)	−0.45^***^ (0.04)	−0.45^***^ (0.04)	−0.45^***^ (0.04)	−0.45^***^ (0.04)
Age (std.)	0.20^***^ (0.02)	0.20^***^ (0.02)	0.20^***^ (0.02)	0.20^***^ (0.02)	0.20^***^ (0.02)	0.20^***^ (0.02)	0.20^***^ (0.02)
Age (std.) × Female	−0.09^***^ (0.01)	−0.09^***^ (0.01)	−0.09^***^ (0.01)	−0.09^***^ (0.01)	−0.09^***^ (0.01)	−0.09^***^ (0.01)	−0.09^***^ (0.01)
Years of education (std.)	0.13^***^ (0.01)	0.13^***^ (0.01)	0.13^***^ (0.01)	0.13^***^ (0.01)	0.13^***^ (0.01)	0.13^***^ (0.01)	0.13^***^ (0.01)
Living with partner	0.16^***^ (0.01)	0.16^***^ (0.01)	0.16^***^ (0.01)	0.16^***^ (0.01)	0.16^***^ (0.01)	0.16^***^ (0.01)	0.16^***^ (0.01)
Good mood scale	0.03^**^ (0.01)	0.03^**^ (0.01)	0.03^**^ (0.01)	0.03^**^ (0.01)	0.03^**^ (0.01)	0.03^**^ (0.01)	0.03^**^ (0.01)
Frequency of social encounters (std.)	0.06^***^ (0.01)	0.06^***^ (0.01)	0.06^***^ (0.01)	0.06^***^ (0.01)	0.06^***^ (0.01)	0.06^***^ (0.01)	0.06^***^ (0.01)
Overall religiosity	−0.08^***^ (0.01)	−0.08^***^ (0.01)	−0.08^***^ (0.01)	−0.08^***^ (0.01)	−0.08^***^ (0.01)	−0.08^***^ (0.01)	−0.08^***^ (0.01)
**COUNTRY LEVEL**							
Openness to Change (vs. Conservation) (std.)		−0.05 (0.04)	−0.06 (0.04)	−0.05 (0.04)	−0.06 (0.04)	−0.07 (0.04)	−0.03 (0.04)
Self-Transcendence (vs. Self-Enhancement) (std.)		0.07 (0.04)	0.05 (0.04)	0.05 (0.04)	0.05 (0.04)	0.07 (0.04)	0.05 (0.04)
**CROSS-LEVEL INTERACTIONS**							
Openness to Change (vs. Conservation) (individual) × Openness to Change (vs. Conservation) (country)				−0.01 (0.01)^*†*^			
x Self-Transcendence (vs. Self-Enhancement) (country)					−0.0004 (0.01)		
Self-Transcendence (vs. Self-Enhancement) (individual) × Self-Transcendence (vs. Self-Enhancement) (country)						−0.01^*^ (0.01)	
× Openness to Change (vs. Conservation) (country)							−0.03^***^ (0.01)
Constant	0.20^***^ (0.05)	0.20^***^ (0.05)	0.21^***^ (0.06)	0.20^***^ (0.06)	0.21^***^ (0.06)	0.21^***^ (0.06)	0.20^***^ (0.05)
**RANDOM VARIANCES**							
Openness to Change (vs. Conservation)			0.001#	0.002#	0.001#	0.001#	0.001#
Self-Transcendence (vs. Self-Enhancement)			0.001#	0.001#	0.001#	0.001#	0.0001
Female	0.026#	0.026#	0.031#	0.031#	0.031#	0.031#	0.031#
Age (std.)	0.009#	0.009#	0.01#	0.01#	0.01#	0.01#	0.01#
Intercepts	0.059#	0.058#	0.068#	0.065#	0.068#	0.068#	0.059#
Residuals	0.76#	0.76#	0.757#	0.757#	0.757#	0.758#	0.757#
**MODEL FIT**							
Deviance	97,275	97,272	97,189	97,189	97,189	97,186	97,183
Number of parameters	18	20	29	30	30	30	30
AIC – Akaike information criterion	97,311	97,312	97,247	97,249	97,249	97,246	97,243
BIC – Bayesian information criterion	97,465	97,483	97,494	97,504	97,505	97,501	97,498

† *p < 0.10*;

* *p < 0.05*;

** *p < 0.01*;

*** *p < 0.001*.

# *significant at p < 0.05 based on bootstrapped confidence intervals*.

*N = 36,928 observations in 21 countries*.

At the individual level, the Openness to Change (vs. Conservation) value dimension showed a strong positive association with frequency of drinking, even after including the control variables. This supported our hypothesis H1. At the same time, Self-Transcendence (vs. Self-Enhancement) did not show a fixed effect that was different from zero. Tables S3, S6 in the Supplementary Materials mirror these results with the 10 value types and 4 higher-order values: the largest positive effect on the frequency of drinking was found with Openness to Change (i.e., Hedonism and Self-Direction), and Conservation values (i.e., Tradition, Security, and Conformity) all showed significant negative effects. However, results for the higher-order values of Self-Transcendence and Self-Enhancement and their respective value types were not significantly associated with drinking.

The control variables showed effects that were consistent with the literature: respondents who were male, older, more educated, and lived with a partner drank more often. Frequency of social encounters and higher levels of wellbeing (i.e., lower depression) were also positively related with the frequency of drinking while religiosity was negatively associated with it. The interaction of age and female gender was significant and negative, indicating that the gender gap in frequency of drinking increases with age.

At the country level, the multilevel results showed that the coefficients for both value dimensions were non-significant, indicating that hypothesis H2 and H3 were not supported. These results were replicated when conducting an analysis with the ten basic values and four higher-order values at the country level (see Tables S1, S5).

Model M3 showed that the variance of random effects was significant for both value dimensions, as evidenced by the bootstrapped confidence intervals of these variances. A decrease in the Akaike information criterion (AIC) in models with and without random effects showed that including the random effects of the two value dimensions was a reasonable extension of the model. Likelihood ratio tests computed as the difference between deviances of these two models showed a significant increase in model fit (82.95, 9 degrees of freedom, significant at *p* < 0.001). At the same time, the more conservative Bayesian information criterion slightly increased, indicating that the increase in model fit is not large relative to its increase in complexity. Nevertheless, the variance of random effects was significant, so we tested whether country-level values could explain the slope variance.

**Figure 1:**
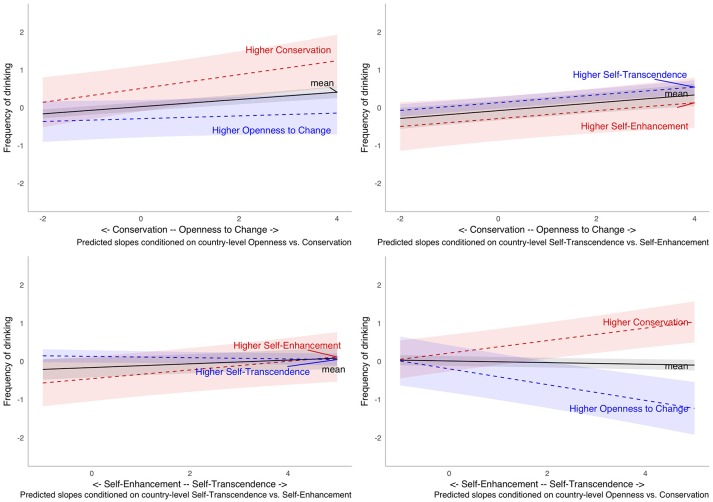
Predicted interaction effects of individual and country-level value dimensions (lines correspond to average country-level values and mean plus/minus two standard deviations of the same value).

Models M4 to M7 (Table 3) tested interactions between country- and individual-level value dimensions in predicting frequency of drinking. The interaction between individual-level and country-level Openness to Change (vs. Conservation) was only marginally significant (*p* < 0.1, *t* = 1.7). The interaction of individual Openness to Change (vs. Conservation) with country-level Self-Transcendence (vs. Self-Enhancement) was not significant. In contrast, the effects of Self-Transcendence (vs. Self-Enhancement) were significantly moderated by both country-level value dimensions. Even though there were significant cross-level effects, the pattern of results did not confirm our Hypothesis (H4), but showed trends that were opposite to what we expected, as illustrated in Figure 1.

The figure shows that the slope for individual-level Openness to Change (vs. Conservation) is more positively associated with frequent drinking in countries valuing Conservation compared to those countries endorsing Openness to Change. The effects of Self-Transcendence (vs. Self-Enhancement) even change their nature depending on the country-level value dimensions: in countries with higher Openness to Change or Self-Transcendence, the effects of individual-level Self-Transcendence (vs. Self-Enhancement) are negative while in countries with higher Conservation or Self-Enhancement they are positive.

Looking at the interactions of the ten basic and the four higher-order values listed in the Supplementary Material (Tables S4, S6), we can see that interactions between personal values belonging to Self-Enhancement or Self-Transcendence and country-level values show a fairly consistent pattern. For example, individual-level Universalism has positive effects on the frequency of drinking in countries that emphasize Security and Tradition values, and less positive or even negative slopes in countries that value Self-Direction and Hedonism. Interactions between the individual basic values belonging to the Openness to Change (vs. Conservation) dimension and country-level values are somewhat less consistent and show more non-significant coefficients.

The analysis using the four higher-order values further supports the identified pattern. In addition, it suggests that the interaction between individual- and country-level Self-Transcendence (vs. Self-Enhancement) is mostly due to individual Self-Enhancement and country-level Self-Transcendence. The analysis also shows a weak positive interaction of both individual Openness and Conservation with country-level Self-Transcendence, demonstrating more positive and less negative effects, respectively, in countries scoring high on Self-Transcendence. Overall, the specific interactions support the more general results for the value dimensions reported above, highlighting some minor specifics.

## Discussion and conclusion

The current study examined how values relate to the frequency of drinking and how this values-drinking behavior link may be moderated by culture across 21 European countries. We found some support for our hypotheses. As expected, the individual-level value dimension Openness to Change (vs. Conservation) was positively related to the frequency of drinking. This is consistent with previous research showing that individuals who value pleasure and enjoyment also exhibit more frequent drinking, whereas those valuing conformity and security drink alcohol less frequently (cf. Table 1).

Country-level correlations demonstrated a positive association between frequent alcohol consumption and Openness to Change (vs. Conservation) as well as Self-Transcendence (vs. Self-Enhancement) cultural values. This is consistent with the empirical evidence showing that individuals residing in countries that value Autonomy, Egalitarianism, and Harmony drink more frequently than individuals residing in countries that value Embeddedness and Hierarchy (Inman et al., 2017). However, in the multilevel regression models, after controlling for sample composition, the associations between the two value dimensions and drinking were found to be non-significant. One explanation is that, in the given sample of countries, country differences in average frequency of drinking are related to differences in population structure, including gender and age. Another reason might also be the small number of countries in the sample and, therefore, limited variability of both the criterion variable (only 8% of the variance was at the country level) and independent variables (country-level values). While our hypotheses about the direct effects of values at the individual level were supported, the country-level hypotheses were not.

Moreover, our results on how cultural values may moderate the values-drinking link were contrary to what we expected. Following previous theorizing in the literature, we reasoned that cultures differ in the extent to which they encourage individuals to act upon their personal values (Roccas and Sagiv, 2010). Cultures that promote intrinsic motivations through emphasizing Openness to Change and Self-Transcendence values should encourage people to express their personal values in all kinds of value-expressive behaviors (including drinking), therefore leading to a stronger link between personal values and behavior. In contrast, cultures that promote extrinsic motivations through emphasizing Conservation and Self-Enhancement values should prevent them from expressing their values in general and encourage them to follow normatively prescribed behaviors, i.e., to abstain from frequent drinking, regardless of their values. Therefore, the link between personal values and behavior should be weaker in these cultures.

However, the data did not support these hypotheses, but showed the opposite pattern to what we theorized: there was a positive association between personal Self-Transcendence (vs. Self-Enhancement) values and frequency of drinking in countries putting higher importance on extrinsic motivations (i.e., Conservation or Self-Enhancement values), while this link was less positive or even negative in countries valuing intrinsic motivations (i.e., Openness to Change or Self-Transcendence values). The marginally significant and negative interaction between individual- and country-level Openness to Change (vs. Conservation) also supported this pattern.

We suggest two possible mechanisms that could explain these findings. First, in countries that emphasize extrinsic motivation, social approval and rewards may be provided to more conservative individuals and to those who emphasize Self-Enhancement, so that they are encouraged to express their values in the form of less frequent drinking. Indeed, Conservation values directly involve conformity, whereas Self-Enhancement values involve conformism through motivation for social approval (Kajonius et al., 2015). In contrast, countries that emphasize intrinsic motivations through Openness and Self-Transcendence values keep normative pressure at relatively low levels; therefore expression of values through drinking is neither rewarded nor sanctioned, leading to the less positive or even negative links with drinking behavior.

Second, self-affirmation processes (Steele, 1988) may explain why individuals with higher Openness to Change and Self-Transcendence values drink more frequently in more conservative societies (and by trend in the ones oriented toward Self-Enhancement values). Their self-concept includes personal freedom and adherence to universalistic norms (beyond their social surroundings). However, this self-concept may be perceived to be under threat in a social context in which societal norms require the opposite. For these individuals, drinking alcohol may be an effective way to express their individual freedom from social norms and expectations that demand abstention from frequent drinking. Engaging in activities that promote the values that are important to an individual can promote self-integrity. Promoting one's values through these behaviors can affirm the individual and consequently reduce the perceived threat (Sherman and Cohen, 2006). Hence, engaging in drinking behavior in conservative cultures may serve to remind individuals of the Openness to Change and Self-Transcendence values by which they define themselves and try to live their lives. Both of these mechanisms are inherent consequences of more restrictive and socially “tight” cultures, as is the case in Conservation and Self-Enhancement cultures. Finally, in some ways, drinking behavior could be seen as non-normative behavior which polarizes the population in conservative societies, dividing them into those who follow these norms and those who protest against it as a form of self-affirmation.

In contrast, in more open and more “socially loose” societies, there are no strict norms or expectations and there are no social rewards for more or less drinking. This also means that the prevalent social and cultural norms are not perceived as a potential threat to one's self-concept. Therefore, expressing one's Openness to Change or Self-Enhancement values through more frequent drinking is not socially or psychologically beneficial in these cultures. It is noteworthy that the value-drinking link was not just attenuated in countries valuing more intrinsic motivations, but even became negative in the specific case of individual-level endorsement of Self-Transcendence (vs. Self-Enhancement) in Openness to Change cultures. A possible explanation (though not an exclusive one) is that differences in the value-drinking links evolved due to different value instantiations (i.e., context-specific expressions of values, see Hanel et al., 2017) that depend on the context. Instantiation of personal Self-Transcendence values in societies scoring high on Openness might find its expression in a broader set of behaviors, such as engaging in helping behavior or caring about nature. In contrast, in conservative cultures the expression of Self-Transcendence values might be limited to higher sociability, which in turn leads to more frequent drinking. Future research could more closely examine the phenomenon of value instantiation and the proposed mechanisms that suggest a context-dependent association between values and behavior. Likewise, personal endorsement of Openness to Change values might be instantiated as freedom to drink in more conservative societies and as freedom to be involved in activities other than drinking in societies that value Openness. Different instantiations could also be related to opportunities provided by different societies in regard to expression of values: in conservative societies, a variety of opportunities tend to be suppressed and it is likely that one of the few options available to express Openness or Hedonism is to break the social rules and drink. On the other hand, societies with higher emphasis on Openness to Change offer more options for instantiating one's values, of which drinking is only one.

Overall, the evidence from our study challenged the general idea put forward in the literature that more “socially tight” cultures that emphasize extrinsic motivations place constraints on acting upon one's values—at least in the particular case of drinking frequency behavior. The opposite seems to be the case; these cultures seem to strengthen the link between personal values and frequency of drinking, or even transform it from negative to positive. In a way, this supports the findings of Kuntsche et al. (2014), who show that among older students in Northern Europe (usually scoring higher on Openness and lower on Conservation values), the link between frequency of drinking with enjoyment and conformity motives was weaker than in Southern Europe (scoring higher on Conservation and lower on Openness values). Our results offer a new theoretical perspective by suggesting that freedom to express one's personal values does not always lead to a stronger value-behavior link.

Our study has some limitations. First, it is limited to one dimension of drinking, namely the frequency of drinking, which is more related to individual preferences, lifestyle, and cultural patterns of drinking, rather than to risky health behaviors and alcohol abuse. It is reasonable to expect that binge drinking or degrees of drunkenness have different correlates and distributions across cultures. Second, as mentioned above, the sample was limited to 21 European countries, and these countries were not selected randomly. This limits the generalization of the results to a larger set of countries. The relatively small sample size also underpowers country-level effects, restricting the inclusion of covariates at the country level and making them less precise than they could be in a larger sample. This might also explain why one of the cross-level interaction effects was only marginally significant. However, the overall pattern of results—also supported by the additional analyses reported in the Supplementary Materials—corroborates our interpretation of the findings. Future research with data from more countries could establish whether the effects of cultural values are reproducible outside Europe and whether they change after controlling for the relevant country characteristics, such as climate or prevalent religion. Third, causality between values and drinking behavior is not guaranteed in our study, as all the key variables were measured at the same time. It is possible that more frequent drinking leads to less stress or more emphasis on Hedonism values. However, as is the case with most behaviors, the direction of causality from values to behavior is more plausible (for a review, see Maio, 2016). These relations are less obvious at the country level, where the opposite direction of causality is possible, as well as some third variable that may determine these relations. Fourth, we studied self-reported behaviors, and since the frequency of drinking can be a sensitive issue, respondents could have underreported their drinking behavior. Fifth, we examined the direct link between values and the frequency of drinking, although these relations might be mediated by many variables, such as attitudes to drinking, drinking intentions, or—as we suggested above—value instantiations.

Despite these limitations, the current study makes an important contribution to the literature. It is the first study to report cross-country differences in value-drinking links. It sheds light on how the value-drinking behavior link may be moderated by the cultural context. And, most importantly, it identifies a number of novel results suggesting a set of possible explanations that can shed more light on explaining drinking patterns across nations, taking into account not only individual characteristics but also the cultural contexts in which individuals live. Ultimately, this study suggests that the strength of the value-behavior link differs across cultures, yet in a way that is more complex than was previously assumed. This opens up new possibilities for research on values and behavior in a cross-cultural context.

## Data availability statement

The European Social Survey data is freely available online on http://europeansocialsurvey.org. All the codes that produced models, tables, and figures are available at https://osf.io/94357.

## Author contributions

MR conducted the statistical analysis and wrote the first draft of the paper. C-MV suggested the idea of the paper, wrote Introduction, and contributed to the writing of all the other parts of the paper.

### Conflict of interest statement

The authors declare that the research was conducted in the absence of any commercial or financial relationships that could be construed as a potential conflict of interest.
