# Supplementary material for: The Link Between Personal Values and Frequency of Drinking Depends on Cultural Values: A Cross-Level Interaction Approach
Source: Front Psychol. 2018 Aug 7;9:1379. doi: 10.3389/fpsyg.2018.01379 (PMC6090463; doi:10.3389/fpsyg.2018.01379)

Supplementary Material

The Link Between Personal Values and Frequency of Drinking Depends on Cultural Values: A Cross-Level Interaction Approach

*Maksim Rudnev^1,2^, Christin-Melanie Vauclair^2^*

^1^ National Research University Higher School of Economics

^2^ Instituto Universitário de Lisboa (ISCTE-IUL), CIS-IUL, Lisboa, Portugal

*** Correspondence**

[maksim.rudnev@gmail.com](mailto:maksim.rudnev@gmail.com)

[Table S1. Sample characteristics by country (variables before standardization) 2](#_Toc520206648)

[Table S2. Measurement invariance test of CES Depression Scale across 21 countries: model fit indices of multiple group confirmatory factor analysis with different sets of the cross-group constraints 3](#_Toc520206649)

[Table S3. Multilevel regressions involving 10 basic values as predictors of frequency of drinking at the individual and country levels^a^ 4](#_Toc520206650)

[Table S4. Interaction effects between ten basic values at the individual and country levels in models predicting frequency of drinking. Each cell contains a regression coefficient from different models. Models included country-level effect of value, interaction with one of the individual-level values (shown in the table), and individual-level predictors listed in Table S3.^a^ 6](#_Toc520206651)

[Table S5. Multilevel regression of higher order values predicting frequency of drinking^a^ 7](#_Toc520206652)

[Table S6. Cross-level interactions between random effects and country-level four higher order values^a^ 9](#_Toc520206653)

[Table S7. Multilevel regressions involving 10 basic values as predictors of frequency of drinking at the individual and country levels^a^ 10](#_Toc520206654)

[Table S8. Interaction effects between ten basic values at the individual and country levels in models predicting frequency of drinking. Each cell contains a regression coefficient from different models. Models included country-level effect of value, interaction with one of the individual-level values (shown in the table), and individual-level predictors listed in Table S7.^a^ 12](#_Toc520206655)

[Table S9. Multilevel regression of higher order value dimensions predicting frequency of drinking^a^ 13](#_Toc520206656)

[Table S10. Cross-level interactions between random effects and country-level four higher order values^a^ 15](#_Toc520206657)

[Figure S1. Frequency of alcohol consumption across 21 European countries 16](#_Toc520206658)

# Table S1. Sample characteristics by country (variables before standardization)

|  | Number of  respondents | Average  age | Percent  female | Openness to Change  (vs. Conservation) | Self-Transcendence  (vs. Self-Enhancement) | Average  frequency of drinking | Correlation between frequency and Openness to Change (vs. Conservation) | Correlation between frequency and Self-Transcendence  (vs. Self-Enhancement) |
| --- | --- | --- | --- | --- | --- | --- | --- | --- |
|  | | | | | | | | |
| Austria | 1795 | 49.22 | 0.52 | -0.29 | 1.16 | 3.82 | 0.14*** | -0.06* |
| Belgium | 1769 | 46.94 | 0.49 | -0.13 | 1.36 | 4.12 | 0.13*** | 0.05* |
| Czech Rep. | 2148 | 46.80 | 0.53 | -0.41 | 0.68 | 3.36 | 0.06** | 0.00 |
| Denmark | 1502 | 48.13 | 0.48 | 0.16 | 1.48 | 4.41 | 0.07** | -0.06* |
| Estonia | 2051 | 50.32 | 0.59 | -0.54 | 1.43 | 3.10 | 0.33*** | -0.11*** |
| Finland | 2087 | 51.31 | 0.51 | -0.11 | 1.96 | 3.52 | 0.11*** | -0.12*** |
| France | 1917 | 49.88 | 0.52 | -0.07 | 1.86 | 4.15 | 0.11*** | 0.04 |
| Germany | 3045 | 49.90 | 0.49 | -0.07 | 1.74 | 4.18 | 0.10*** | -0.08*** |
| Hungary | 1698 | 49.87 | 0.57 | -0.28 | 0.80 | 2.51 | 0.15*** | -0.07** |
| Ireland | 2390 | 49.39 | 0.54 | -0.41 | 1.09 | 3.52 | 0.12*** | -0.01 |
| Israel | 2562 | 47.65 | 0.55 | -0.36 | 0.85 | 2.24 | 0.21*** | 0.01 |
| Lithuania | 2250 | 49.73 | 0.61 | -0.70 | 0.33 | 2.74 | 0.20*** | -0.11*** |
| Netherlands | 1919 | 50.74 | 0.55 | 0.10 | 1.33 | 4.19 | 0.11*** | -0.04 |
| Norway | 1436 | 46.77 | 0.47 | -0.16 | 1.49 | 3.69 | 0.13*** | -0.02 |
| Poland | 1615 | 47.30 | 0.54 | -1.02 | 1.11 | 2.99 | 0.30*** | -0.11*** |
| Portugal | 1265 | 52.90 | 0.55 | -0.31 | 1.30 | 3.74 | 0.07** | -0.01 |
| Slovenia | 1224 | 49.58 | 0.54 | -0.38 | 1.06 | 3.51 | 0.09** | 0.00 |
| Spain | 1925 | 48.54 | 0.49 | -0.59 | 1.91 | 3.85 | 0.12*** | 0.01 |
| Sweden | 1791 | 49.70 | 0.5 | 0.20 | 1.87 | 3.81 | 0.10*** | -0.07** |
| Switzerland | 1532 | 47.36 | 0.50 | 0.11 | 1.38 | 4.22 | 0.08** | -0.03 |
| United Kingdom | 2264 | 52.20 | 0.55 | -0.33 | 1.56 | 3.96 | 0.14*** | -0.01 |
| Total | 40185 | 49.28 | 0.53 | -0.27 | 1.33 | 3.58 | 0.15 | -0.04 |
|  |  |  |  |  |  |  |  |  |

*Note.* ^*^p<0.05; ^**^p<0.01; ^***^p<0.001

# Table S2. Measurement invariance test of CES Depression Scale across 21 countries: model fit indices of multiple group confirmatory factor analysis with different sets of the cross-group constraints

|  | Degrees of freedom | Chi-square | Chi-square difference | Significance of chi-square difference | CFI | RMSEA | Difference CFI | Difference RMSEA |
| --- | --- | --- | --- | --- | --- | --- | --- | --- |
| Configural invariance model | 399 | 4292.8 |  |  | 0.96 | 0.072 |  |  |
| Metric invariance (loadings are fixed across groups except for "could not get going" item) | 519 | 5325.6 | 1032.8 | <.001 | 0.951 | 0.07 | 0.009 | 0.002 |
| Scalar invariance (intercepts and loadings are fixed, except for "could not get going" item) | 659 | 11417.1 | 6091.5 | <.001 | 0.89 | 0.094 | 0.061 | 0.023 |

*Note.* Measurement invariance was assessed using Chen (2008) criterion, namely, the difference in CFI and RMSEA between the two nested models should not be higher than 0.01. This criterion holds for metric measurement invariance. Chi-square criterion and its difference is not applicable in large samples, which is the case (N=37,121).

# Table S3. Multilevel regressions involving 10 basic values as predictors of frequency of drinking at the individual and country levels^a^

|  | (1) | (2) | (3) | (4) | (5) | (6) | (7) | (8) | (9) | (10) | |
| --- | --- | --- | --- | --- | --- | --- | --- | --- | --- | --- | --- |
|  | | | | | | | | | | | |
| ***Individual level*** |  |  |  |  |  |  |  |  |  |  | |
| Security | -0.05^***^ (0.01) | -0.05^***^ (0.01) | -0.05^***^ (0.01) | -0.05^***^ (0.01) | -0.05^***^ (0.01) | -0.05^***^ (0.01) | -0.05^***^ (0.01) | -0.06^***^ (0.01) | -0.05^***^ (0.01) | -0.05^***^ (0.01) | |
| Conformity | -0.08^***^ (0.01) | -0.08^***^ (0.01) | -0.08^***^ (0.01) | -0.08^***^ (0.01) | -0.08^***^ (0.01) | -0.08^***^ (0.01) | -0.08^***^ (0.01) | -0.08^***^ (0.01) | -0.08^***^ (0.01) | -0.08^***^ (0.01) | |
| Tradition | -0.07^***^ (0.01) | -0.07^***^ (0.01) | -0.07^***^ (0.01) | -0.07^***^ (0.01) | -0.07^***^ (0.01) | -0.07^***^ (0.01) | -0.07^***^ (0.01) | -0.07^***^ (0.01) | -0.07^***^ (0.01) | -0.07^***^ (0.01) | |
| Benevolence | 0.01 (0.01) | 0.01 (0.01) | 0.01 (0.01) | 0.004 (0.01) | 0.005 (0.01) | 0.004 (0.01) | 0.005 (0.01) | 0.01 (0.01) | 0.005 (0.01) | 0.005 (0.01) | |
| Universalism | -0.02 (0.02) | -0.02 (0.02) | -0.02 (0.02) | -0.02 (0.02) | -0.02 (0.02) | -0.02 (0.02) | -0.02 (0.02) | -0.02 (0.02) | -0.02 (0.02) | -0.02 (0.02) | |
| Self-Direction | 0.03^**^ (0.01) | 0.03^**^ (0.01) | 0.03^**^ (0.01) | 0.03^**^ (0.01) | 0.03^**^ (0.01) | 0.03^**^ (0.01) | 0.03^**^ (0.01) | 0.03^**^ (0.01) | 0.03^**^ (0.01) | 0.03^**^ (0.01) | |
| Stimulation | 0.01 (0.01) | 0.01 (0.01) | 0.01 (0.01) | 0.01 (0.01) | 0.01 (0.01) | 0.01 (0.01) | 0.01 (0.01) | 0.01 (0.01) | 0.01 (0.01) | 0.01 (0.01) | |
| Hedonism | 0.19^***^ (0.01) | 0.19^***^ (0.01) | 0.19^***^ (0.01) | 0.19^***^ (0.01) | 0.19^***^ (0.01) | 0.19^***^ (0.01) | 0.19^***^ (0.01) | 0.19^***^ (0.01) | 0.19^***^ (0.01) | 0.19^***^ (0.01) | |
| Achievement | -0.01 (0.01) | -0.01 (0.01) | -0.01 (0.01) | -0.01 (0.01) | -0.01 (0.01) | -0.01 (0.01) | -0.01 (0.01) | -0.01 (0.01) | -0.01 (0.01) | -0.01 (0.01) | |
| Power | -0.01 (0.01) | -0.01 (0.01) | -0.01 (0.01) | -0.01 (0.01) | -0.01 (0.01) | -0.01 (0.01) | -0.01 (0.01) | -0.01 (0.01) | -0.01 (0.01) | -0.01 (0.01) | |
| Gender (female) | -0.92^***^ (0.07) | -0.92^***^ (0.07) | -0.92^***^ (0.07) | -0.92^***^ (0.07) | -0.92^***^ (0.07) | -0.92^***^ (0.07) | -0.92^***^ (0.07) | -0.92^***^ (0.07) | -0.92^***^ (0.07) | -0.92^***^ (0.07) | |
| Age (std.) | 0.42^***^ (0.04) | 0.42^***^ (0.04) | 0.42^***^ (0.04) | 0.42^***^ (0.04) | 0.42^***^ (0.04) | 0.42^***^ (0.04) | 0.42^***^ (0.04) | 0.42^***^ (0.04) | 0.42^***^ (0.04) | 0.42^***^ (0.04) | |
| Female x Age (std.) | -0.17^***^ (0.02) | -0.17^***^ (0.02) | -0.17^***^ (0.02) | -0.17^***^ (0.02) | -0.17^***^ (0.02) | -0.17^***^ (0.02) | -0.17^***^ (0.02) | -0.17^***^ (0.02) | -0.17^***^ (0.02) | -0.17^***^ (0.02) | |
| Years of education (std.) | 0.26^***^ (0.01) | 0.26^***^ (0.01) | 0.26^***^ (0.01) | 0.26^***^ (0.01) | 0.26^***^ (0.01) | 0.26^***^ (0.01) | 0.26^***^ (0.01) | 0.26^***^ (0.01) | 0.26^***^ (0.01) | 0.26^***^ (0.01) | |
| Living with partner | 0.32^***^ (0.02) | 0.32^***^ (0.02) | 0.32^***^ (0.02) | 0.32^***^ (0.02) | 0.32^***^ (0.02) | 0.32^***^ (0.02) | 0.32^***^ (0.02) | 0.32^***^ (0.02) | 0.32^***^ (0.02) | 0.32^***^ (0.02) | |
| Depression mood scale, reversed | 0.05^*^ (0.02) | 0.05^*^ (0.02) | 0.05^*^ (0.02) | 0.05^*^ (0.02) | 0.05^*^ (0.02) | 0.05^*^ (0.02) | 0.05^*^ (0.02) | 0.05^*^ (0.02) | 0.05^*^ (0.02) | 0.05^*^ (0.02) | |
| Frequency of social meetings (std.) | 0.12^***^ (0.01) | 0.12^***^ (0.01) | 0.12^***^ (0.01) | 0.12^***^ (0.01) | 0.12^***^ (0.01) | 0.12^***^ (0.01) | 0.12^***^ (0.01) | 0.12^***^ (0.01) | 0.12^***^ (0.01) | 0.12^***^ (0.01) | |
| Overall religiosity | -0.16^***^ (0.01) | -0.16^***^ (0.01) | -0.16^***^ (0.01) | -0.16^***^ (0.01) | -0.16^***^ (0.01) | -0.16^***^ (0.01) | -0.16^***^ (0.01) | -0.16^***^ (0.01) | -0.16^***^ (0.01) | -0.16^***^ (0.01) | |
| ***Country level*** |  |  |  |  |  |  |  |  |  |  | |
| Security | 0.17 (0.30) |  |  |  |  |  |  |  |  |  | |
| Conformity |  | 0.05 (0.36) |  |  |  |  |  |  |  |  | |
| Tradition |  |  | 0.49 (0.43) |  |  |  |  |  |  |  | |
| Benevolence |  |  |  | 0.56 (0.33) |  |  |  |  |  |  | |
| Universalism |  |  |  |  | 0.52 (0.42) |  |  |  |  |  | |
| Self-Direction |  |  |  |  |  | 0.94^*^ (0.48) |  |  |  |  | |
| Stimulation |  |  |  |  |  |  | -0.88 (0.54) |  |  |  | |
| Hedonism |  |  |  |  |  |  |  | -0.46 (0.24) |  |  | |
| Achievement |  |  |  |  |  |  |  |  | -0.41 (0.28) |  | |
| Power |  |  |  |  |  |  |  |  |  | -0.28 (0.25) | |
| Constant | 3.96^***^ (0.18) | 4.04^***^ (0.14) | 4.02^***^ (0.13) | 3.63^***^ (0.26) | 3.73^***^ (0.27) | 3.67^***^ (0.22) | 3.41^***^ (0.40) | 3.95^***^ (0.15) | 3.84^***^ (0.18) | 3.78^***^ (0.27) | |
|  | | | | | | | | | | | |
| AIC | 148,200 | 148,200 | 148,200 | 148,198 | 148,199 | 148,198 | 148,199 | 148,198 | 148,199 | 148,199 | |
| BIC | 148,430 | 148,430 | 148,429 | 148,428 | 148,429 | 148,428 | 148,429 | 148,428 | 148,429 | 148,429 | |
| Variance of intercepts | 0.239 | 0.23 | 0.232 | 0.202 | 0.213 | 0.188 | 0.242 | 0.283 | 0.202 | 0.207 | |
| Variance of female | 0.099 | 0.1 | 0.099 | 0.1 | 0.1 | 0.1 | 0.099 | 0.099 | 0.1 | 0.1 | |
| Variance of age | 0.036 | 0.036 | 0.036 | 0.036 | 0.036 | 0.036 | 0.036 | 0.036 | 0.036 | 0.036 | |
| Variance of residuals | 3.036 | 3.036 | 3.036 | 3.036 | 3.036 | 3.036 | 3.036 | 3.036 | 3.036 | 3.036 | |
|  | | | | | | | | | | | |
| ^*^p<0.05; ^**^p<0.01; ^***^p<0.001. | | | | | | | | | | |  |

^a^ Value indices at the individual level are non-ipsatized simple averages of two or three value items; value indices at the country level were computed in three steps: first, we used individual-level value simple averages; second, we ipsatized them by subtracting an individual mean on all the value items from each value index; third, resulting ipsatized value indices were averaged at the country level. Ipsatization was deemed necessary because (1) it corrects for response style, (2) it provides scores that reflect relative rather than absolute importance of values; relative importance is the one that was theorized and shown to guide behavior, (3)virtually all of the studies demonstrating the relations between values and behavior used ipsatized scores, in contrast, we do not know any study of value-behavior or value-attitudes links that used non-ipsatized value scores. Considering recent discussions about ipsatized scores in values research (*Borg, I., & Bardi, A. (2016). Should ratings of the importance of personal values be centered? Journal of Research in Personality, 63, 95-101*), we also report the model results with non-ipsatized country-level value scores in Tables S7-S10.

# Table S4. Interaction effects between ten basic values at the individual and country levels in models predicting frequency of drinking. Each cell contains a regression coefficient from different models. Models included country-level effect of value, interaction with one of the individual-level values (shown in the table), and individual-level predictors listed in Table S3.^a^

|  | **Country-level values** | | | | | | | | | | |
| --- | --- | --- | --- | --- | --- | --- | --- | --- | --- | --- | --- |
| **Individual-level effects** | Security | Conformity | Tradition | Benevo-lence | Univer-salism | Self-Direction | Stimulation | Hedonism | Achieve-ment | Power |  |
| Security | -0.03(0.05) | 0.03(0.06) | 0.04(0.08) | 0.04(0.06) | 0.04(0.07) | 0.10(0.09) | 0.31(0.09)** | -0.03(0.05) | -0.09(0.05) | -0.03(0.04) |  |
| Conformity | -0.06(0.06) | -0.07(0.08) | 0.07(0.09) | 0.18(0.06)* | 0.22(0.08)* | 0.31(0.09)** | 0.12(0.12) | -0.03(0.05) | -0.16(0.05)** | -0.12(0.05)* |  |
| Tradition | 0.03(0.05) | -0.04(0.07) | 0.12(0.08) | 0.04(0.06) | 0.07(0.08) | 0.08(0.09) | 0.12(0.10) | -0.08(0.05) | -0.05(0.05) | -0.02(0.05) |  |
| Benevolence | 0.24(0.06)** | -0.01(0.09) | 0.28(0.09)** | -0.08(0.07) | -0.01(0.09) | -0.12(0.12) | -0.25(0.14) | -0.09(0.06) | -0.01(0.06) | 0.05(0.06) |  |
| Universalism | 0.23(0.06)** | 0.08(0.08) | 0.28(0.09)** | -0.08(0.07) | -0.05(0.09) | -0.20(0.10)* | -0.24(0.14) | -0.21(0.06)** | 0.00(0.06) | 0.03(0.05) |  |
| Self-Direction | 0.10(0.05)* | -0.02(0.06) | 0.15(0.07)* | -0.02(0.08) | -0.06(0.1) | -0.04(0.08) | -0.18(0.09)* | -0.01(0.04) | -0.04(0.05) | -0.03(0.04) |  |
| Stimulation | 0.01(0.04) | -0.11(0.04)* | 0.02(0.06) | 0.03(0.04) | 0.04(0.06) | 0.03(0.07) | -0.18(0.07)* | 0.04(0.03) | 0.03(0.04) | -0.02(0.03) |  |
| Hedonism | 0.16(0.04)** | -0.10(0.06) | 0.19(0.07)* | 0.03(0.06) | 0.14(0.07)* | -0.03(0.08) | -0.27(0.08)** | -0.05(0.04) | -0.06(0.05) | -0.06(0.04) |  |
| Achievement | -0.08(0.04)* | -0.10(0.05)* | -0.06(0.06) | 0.15(0.04)** | 0.13(0.05)* | 0.23(0.06)** | -0.11(0.08) | 0.05(0.03) | -0.05(0.04) | -0.07(0.03)* |  |
| Power | -0.10(0.06) | -0.11(0.07) | -0.18(0.08)* | 0.13(0.06)* | 0.14(0.08) | 0.36(0.08)** | -0.07(0.11) | 0.06(0.05) | -0.06(0.05) | -0.05(0.05) |  |

*Note:* ^*^p<0.05; ^**^p<0.01.

^a^ See the note to Table S3.

# Table S5. Multilevel regression of higher order values predicting frequency of drinking^a^

|  | (1) | (2) | (3) | (4) | (5) | (6) |
| --- | --- | --- | --- | --- | --- | --- |
|  | | | | | | |
| ***Individual level*** |  |  |  |  |  |  |
| Conservation | -0.12^***^(0.01) | -0.09^***^(0.01) | -0.09^***^(0.01) | -0.09^***^(0.01) | -0.09^***^(0.01) | -0.09^***^(0.01) |
| Openness to Change | 0.12^***^(0.01) | 0.12^***^(0.01) | 0.12^***^(0.01) | 0.12^***^(0.01) | 0.12^***^(0.01) | 0.12^***^(0.01) |
| Self-Enhancement | -0.01 (0.01) | -0.01^*^(0.01) | -0.02 (0.01) | -0.02 (0.01) | -0.02 (0.01) | -0.01 (0.01) |
| Self-Transcendence | -0.02^*^(0.01) | -0.02^*^(0.01) | -0.02 (0.01) | -0.02 (0.01) | -0.02 (0.01) | -0.02 (0.01) |
| Female |  | -0.45^***^(0.04) | -0.45^***^(0.04) | -0.45^***^(0.04) | -0.45^***^(0.04) | -0.45^***^(0.04) |
| Age (std.) |  | 0.20^***^(0.02) | 0.20^***^(0.02) | 0.20^***^(0.02) | 0.20^***^(0.02) | 0.20^***^(0.02) |
| Female x Age (std.) |  | -0.09^***^(0.01) | -0.09^***^(0.01) | -0.09^***^(0.01) | -0.09^***^(0.01) | -0.09^***^(0.01) |
| Education in years (std.) |  | 0.13^***^(0.01) | 0.13^***^(0.01) | 0.13^***^(0.01) | 0.13^***^(0.01) | 0.13^***^(0.01) |
| Partnered |  | 0.16^***^(0.01) | 0.16^***^(0.01) | 0.16^***^(0.01) | 0.16^***^(0.01) | 0.16^***^(0.01) |
| Depression mood scale, reversed |  | 0.03^**^(0.01) | 0.03^**^(0.01) | 0.03^**^(0.01) | 0.03^**^(0.01) | 0.03^**^(0.01) |
| Frequency of social meetings (std.) |  | 0.06^***^(0.01) | 0.06^***^(0.01) | 0.06^***^(0.01) | 0.06^***^(0.01) | 0.06^***^(0.01) |
| Overall religiosity |  | -0.08^***^(0.01) | -0.08^***^(0.01) | -0.08^***^(0.01) | -0.08^***^(0.01) | -0.08^***^(0.01) |
| ***Country level*** |  |  |  |  |  |  |
| Conservation |  |  | 0.05 (0.03) |  |  |  |
| Openness to Change |  |  |  | -0.05 (0.03) |  |  |
| Self-Enhancement |  |  |  |  | -0.04 (0.03) |  |
| Self-Transcendence |  |  |  |  |  | 0.06 (0.03) |
| Constant | 0.16^*^(0.07) | 0.23^***^(0.07) | 0.22^**^(0.07) | 0.22^**^(0.07) | 0.22^***^(0.07) | 0.22^***^(0.07) |
|  | | | | | | |
| AIC | 108,160 | 97,308 | 97,245 | 97,245 | 97,245 | 97,244 |
| BIC | 108,220 | 97,479 | 97,500 | 97,500 | 97,500 | 97,500 |
| Variance of intercepts | 0.066 | 0.059 | 0.062 | 0.065 | 0.058 | 0.061 |
| Variance of Conservation |  |  | 0.003 | 0.003 | 0.003 | 0.003 |
| Variance of Self-Enhancement |  |  | 0.002 | 0.002 | 0.002 | 0.002 |
| Variance of female |  | 0.026 | 0.028 | 0.028 | 0.028 | 0.028 |
| Variance of age |  | 0.009 | 0.010 | 0.010 | 0.010 | 0.010 |
| Variance of residual | 0.884 | 0.760 | 0.758 | 0.758 | 0.758 | 0.758 |
|  | | | | | | |
| *Note.* ^*^p<0.05; ^**^p<0.01; ^***^p<0.001.  ^a^ See the note to Table S3. |  | | | | | |

# Table S6. Cross-level interactions between random effects and country-level four higher order values^a^

| **Individual-level effects** | **Country-level values** | | | |
| --- | --- | --- | --- | --- |
|  | Conservation | Openness to Change | Self-Enhancement | Self-Transcendence |
|  | | | | |
| Conservation | 0.002(0.01) | 0.01(0.01) | -0.02(0.01)* | 0.02(0.01)* |
| Openness to Change | 0.01(0.01) | -0.02(0.01)* | -0.003 (0.01) | 0.02(0.01)* |
| Self-Enhancement | -0.02(0.01)** | 0.01(0.01)* | -0.01(0.01) | 0.02(0.01)** |
| Self-Transcendence | 0.03(0.01)** | -0.04(0.01)*** | 0.01(0.01) | -0.01(0.01) |
| *Note.* ^*^p<0.05; ^**^p<0.01; ^***^p<0.001*.*  ^a^ See the note to Table S3. | | | |  |

# Table S7. Multilevel regressions involving 10 basic values as predictors of frequency of drinking at the individual and country levels^a^

|  | (1) | (2) | (3) | (4) | (5) | (6) | (7) | (8) | (9) | (10) | |
| --- | --- | --- | --- | --- | --- | --- | --- | --- | --- | --- | --- |
|  | | | | | | | | | | |  |
| ***Individual level*** |  |  |  |  |  |  |  |  |  |  | |
| Security | -0.05^***^(0.01) | -0.05^***^(0.01) | -0.05^***^(0.01) | -0.05^***^(0.01) | -0.05^***^(0.01) | -0.05^***^(0.01) | -0.05^***^(0.01) | -0.05^***^(0.01) | -0.05^***^(0.01) | -0.05^***^(0.01) | |
| Conformity | -0.08^***^(0.01) | -0.08^***^(0.01) | -0.08^***^(0.01) | -0.08^***^(0.01) | -0.08^***^(0.01) | -0.08^***^(0.01) | -0.08^***^(0.01) | -0.08^***^(0.01) | -0.08^***^(0.01) | -0.08^***^(0.01) | |
| Tradition | -0.07^***^(0.01) | -0.07^***^(0.01) | -0.07^***^(0.01) | -0.07^***^(0.01) | -0.07^***^(0.01) | -0.07^***^(0.01) | -0.07^***^(0.01) | -0.07^***^(0.01) | -0.07^***^(0.01) | -0.07^***^(0.01) | |
| Benevolence | 0.01 (0.01) | 0.005 (0.01) | 0.005 (0.01) | 0.005 (0.01) | 0.005 (0.01) | 0.005 (0.01) | 0.01 (0.01) | 0.01 (0.01) | 0.005 (0.01) | 0.005 (0.01) | |
| Universalism | -0.02 (0.02) | -0.02 (0.02) | -0.02 (0.02) | -0.02 (0.02) | -0.02 (0.02) | -0.02 (0.02) | -0.02 (0.02) | -0.02 (0.02) | -0.02 (0.02) | -0.02 (0.02) | |
| Self-Direction | 0.03^**^(0.01) | 0.03^**^(0.01) | 0.03^**^(0.01) | 0.03^**^(0.01) | 0.03^**^(0.01) | 0.03^**^(0.01) | 0.03^**^(0.01) | 0.03^**^(0.01) | 0.03^**^(0.01) | 0.03^**^(0.01) | |
| Stimulation | 0.01 (0.01) | 0.01 (0.01) | 0.01 (0.01) | 0.01 (0.01) | 0.01 (0.01) | 0.01 (0.01) | 0.01 (0.01) | 0.01 (0.01) | 0.01 (0.01) | 0.01 (0.01) | |
| Hedonism | 0.19^***^(0.01) | 0.19^***^(0.01) | 0.19^***^(0.01) | 0.19^***^(0.01) | 0.19^***^(0.01) | 0.19^***^(0.01) | 0.19^***^(0.01) | 0.19^***^(0.01) | 0.19^***^(0.01) | 0.19^***^(0.01) | |
| Achievement | -0.01 (0.01) | -0.01 (0.01) | -0.01 (0.01) | -0.01 (0.01) | -0.01 (0.01) | -0.01 (0.01) | -0.01 (0.01) | -0.01 (0.01) | -0.01 (0.01) | -0.01 (0.01) | |
| Power | -0.01 (0.01) | -0.01 (0.01) | -0.01 (0.01) | -0.01 (0.01) | -0.01 (0.01) | -0.01 (0.01) | -0.01 (0.01) | -0.01 (0.01) | -0.01 (0.01) | -0.01 (0.01) | |
| Gender (female) | -0.92^***^(0.07) | -0.92^***^(0.07) | -0.92^***^(0.07) | -0.92^***^(0.07) | -0.92^***^(0.07) | -0.92^***^(0.07) | -0.92^***^(0.07) | -0.92^***^(0.07) | -0.92^***^(0.07) | -0.92^***^(0.07) | |
| Age (std.) | 0.42^***^(0.04) | 0.42^***^(0.04) | 0.42^***^(0.04) | 0.42^***^(0.04) | 0.42^***^(0.04) | 0.42^***^(0.04) | 0.42^***^(0.04) | 0.42^***^(0.04) | 0.42^***^(0.04) | 0.42^***^(0.04) | |
| Female x Age (std.) | -0.17^***^(0.02) | -0.17^***^(0.02) | -0.17^***^(0.02) | -0.17^***^(0.02) | -0.17^***^(0.02) | -0.17^***^(0.02) | -0.17^***^(0.02) | -0.17^***^(0.02) | -0.17^***^(0.02) | -0.17^***^(0.02) | |
| Years of education (std.) | 0.26^***^(0.01) | 0.26^***^(0.01) | 0.26^***^(0.01) | 0.26^***^(0.01) | 0.26^***^(0.01) | 0.26^***^(0.01) | 0.26^***^(0.01) | 0.26^***^(0.01) | 0.26^***^(0.01) | 0.26^***^(0.01) | |
| Living with partner | 0.32^***^(0.02) | 0.32^***^(0.02) | 0.32^***^(0.02) | 0.32^***^(0.02) | 0.32^***^(0.02) | 0.32^***^(0.02) | 0.32^***^(0.02) | 0.32^***^(0.02) | 0.32^***^(0.02) | 0.32^***^(0.02) | |
| Depression mood scale, reversed | 0.05^*^(0.02) | 0.05^*^(0.02) | 0.05^*^(0.02) | 0.05^*^(0.02) | 0.05^*^(0.02) | 0.05^*^(0.02) | 0.05^*^(0.02) | 0.05^*^(0.02) | 0.05^*^(0.02) | 0.05^*^(0.02) | |
| Frequency of social meetings (std.) | 0.12^***^(0.01) | 0.12^***^(0.01) | 0.12^***^(0.01) | 0.12^***^(0.01) | 0.12^***^(0.01) | 0.12^***^(0.01) | 0.12^***^(0.01) | 0.12^***^(0.01) | 0.12^***^(0.01) | 0.12^***^(0.01) | |
| Overall religiosity | -0.16^***^(0.01) | -0.16^***^(0.01) | -0.16^***^(0.01) | -0.16^***^(0.01) | -0.16^***^(0.01) | -0.16^***^(0.01) | -0.16^***^(0.01) | -0.16^***^(0.01) | -0.16^***^(0.01) | -0.16^***^(0.01) | |
| ***Country level*** |  |  |  |  |  |  |  |  |  |  | |
| Security | 0.12 (0.23) |  |  |  |  |  |  |  |  |  | |
| Conformity |  | 0.04 (0.30) |  |  |  |  |  |  |  |  | |
| Tradition |  |  | 0.20 (0.28) |  |  |  |  |  |  |  | |
| Benevolence |  |  |  | 0.32 (0.28) |  |  |  |  |  |  | |
| Universalism |  |  |  |  | 0.32 (0.36) |  |  |  |  |  | |
| Self-Direction |  |  |  |  |  | 0.29 (0.36) |  |  |  |  | |
| Stimulation |  |  |  |  |  |  | -0.23 (0.41) |  |  |  | |
| Hedonism |  |  |  |  |  |  |  | -0.22 (0.21) |  |  | |
| Achievement |  |  |  |  |  |  |  |  | -0.19 (0.20) |  | |
| Power |  |  |  |  |  |  |  |  |  | -0.21 (0.21) | |
| Constant | 3.47^**^(1.07) | 3.86^**^(1.23) | 3.16^**^(1.19) | 2.46 (1.41) | 2.46 (1.75) | 2.70 (1.66) | 4.86^***^(1.47) | 4.92^***^(0.85) | 4.74^***^(0.76) | 4.73^***^(0.71) | |
| AIC | 148,200 | 148,200 | 148,200 | 148,199 | 148,200 | 148,200 | 148,200 | 148,199 | 148,200 | 148,200 | |
| BIC | 148,430 | 148,430 | 148,430 | 148,429 | 148,429 | 148,430 | 148,430 | 148,429 | 148,430 | 148,430 | |
| Variance of intercepts | 0.24 | 0.231 | 0.236 | 0.221 | 0.228 | 0.222 | 0.223 | 0.245 | 0.21 | 0.207 | |
| Variance of female | 0.099 | 0.1 | 0.099 | 0.1 | 0.1 | 0.1 | 0.1 | 0.1 | 0.1 | 0.1 | |
| Variance of age | 0.036 | 0.036 | 0.036 | 0.036 | 0.036 | 0.036 | 0.036 | 0.036 | 0.036 | 0.036 | |
| Variance of residuals | 3.036 | 3.036 | 3.036 | 3.036 | 3.036 | 3.036 | 3.036 | 3.036 | 3.036 | 3.036 | |

*p<0.05; **p<0.01; ***p<0.001.

^a^ The models used non-ipsatized value scores at both levels.

# Table S8. Interaction effects between ten basic values at the individual and country levels in models predicting frequency of drinking. Each cell contains a regression coefficient from different models. Models included country-level effect of value, interaction with one of the individual-level values (shown in the table), and individual-level predictors listed in Table S7.^a^

|  | **Country-level values** | | | | | | | | | | |
| --- | --- | --- | --- | --- | --- | --- | --- | --- | --- | --- | --- |
| **Individual-level effects** | Security | Conformity | Tradition | Benevo-lence | Univer-salism | Self-Direction | Stimulation | Hedonism | Achieve-ment | Power |  |
| Security | -0.06(0.04) | -0.01(0.05) | -0.02(0.05) | 0.00(0.05) | -0.02(0.07) | -0.01(0.06) | 0.05(0.07) | -0.03(0.04) | -0.06(0.03) | -0.04(0.04) |  |
| Conformity | -0.08(0.05) | -0.09(0.06) | -0.01(0.06) | 0.08(0.06) | 0.1(0.08) | 0.06(0.08) | -0.02(0.09) | -0.03(0.04) | -0.09(0.04)** | -0.11(0.04)** |  |
| Tradition | -0.05(0.04) | -0.1(0.05) | -0.02(0.05) | -0.02(0.05) | -0.03(0.07) | -0.05(0.07) | -0.07(0.08) | -0.07(0.04) | -0.06(0.04) | -0.05(0.04) |  |
| Benevolence | 0.13(0.05)** | 0.02(0.08) | 0.17(0.07)** | -0.07(0.06) | -0.07(0.09) | -0.12(0.09) | -0.05(0.10) | -0.14(0.05)** | -0.03(0.05) | 0.00(0.05) |  |
| Universalism | 0.08(0.05) | -0.02(0.07) | 0.13(0.08) | -0.1(0.06) | -0.13(0.08) | -0.16(0.07)** | -0.16(0.09) | -0.13(0.04)** | -0.04(0.05) | -0.02(0.05) |  |
| Self-Direction | 0.13(0.04)** | 0.03(0.05) | 0.12(0.05)** | 0.05(0.04) | 0.05(0.06) | 0.04(0.06) | 0.03(0.09) | 0.02(0.03) | 0.01(0.03) | 0.00(0.03) |  |
| Stimulation | 0.00(0.03) | -0.08(0.04)* | 0.01(0.04) | 0.01(0.04) | 0.02(0.05) | 0.01(0.05) | -0.06(0.06) | 0.02(0.03) | 0.01(0.03) | -0.02(0.03) |  |
| Hedonism | 0.08(0.04)* | -0.12(0.04)** | 0.04(0.05) | -0.01(0.05) | 0.04(0.06) | -0.05(0.06) | -0.15(0.06)** | -0.04(0.03) | -0.05(0.03) | -0.07(0.03)** |  |
| Achievement | -0.08(0.03)** | -0.09(0.04)** | -0.05(0.04) | 0.07(0.04) | 0.06(0.05) | 0.05(0.05) | -0.06(0.06) | 0.02(0.03) | -0.03(0.03) | -0.06(0.03)* |  |
| Power | -0.10(0.04)** | -0.11(0.05)** | -0.11(0.05)** | 0.05(0.05) | 0.05(0.07) | 0.07(0.07) | -0.06(0.08) | 0.02(0.04) | -0.04(0.04) | -0.05(0.04) |  |

*Note:* ^*^p<0.05; ^**^p<0.01.

^a^ The models used non-ipsatized value scores at both levels.

# Table S9. Multilevel regression of higher order value dimensions predicting frequency of drinking^a^

|  | (1) | (2) | (3) | (4) | (5) | (6) |
| --- | --- | --- | --- | --- | --- | --- |
|  | | | | | | |
| ***Individual level*** |  |  |  |  |  |  |
| Conservation | -0.12^***^(0.01) | -0.09^***^(0.01) | -0.09^***^(0.01) | -0.09^***^(0.01) | -0.09^***^(0.01) | -0.09^***^(0.01) |
| Openness to Change | 0.12^***^(0.01) | 0.12^***^(0.01) | 0.12^***^(0.01) | 0.12^***^(0.01) | 0.12^***^(0.01) | 0.12^***^(0.01) |
| Self-Enhancement | -0.01 (0.01) | -0.01^*^(0.01) | -0.02 (0.01) | -0.02 (0.01) | -0.02 (0.01) | -0.02 (0.01) |
| Self-Transcendence | -0.02^*^(0.01) | -0.02^*^(0.01) | -0.02 (0.01) | -0.02 (0.01) | -0.02 (0.01) | -0.02 (0.01) |
| Female |  | -0.45^***^(0.04) | -0.45^***^(0.04) | -0.45^***^(0.04) | -0.45^***^(0.04) | -0.45^***^(0.04) |
| Age (std.) |  | 0.20^***^(0.02) | 0.20^***^(0.02) | 0.20^***^(0.02) | 0.20^***^(0.02) | 0.20^***^(0.02) |
| Female x Age (std.) |  | 0.13^***^(0.01) | 0.13^***^(0.01) | 0.13^***^(0.01) | 0.13^***^(0.01) | 0.13^***^(0.01) |
| Education in years (std.) |  | -0.09^***^(0.01) | -0.09^***^(0.01) | -0.09^***^(0.01) | -0.09^***^(0.01) | -0.09^***^(0.01) |
| Partnered |  | 0.16^***^(0.01) | 0.16^***^(0.01) | 0.16^***^(0.01) | 0.16^***^(0.01) | 0.16^***^(0.01) |
| Depression mood scale, reversed |  | 0.03^**^(0.01) | 0.03^**^(0.01) | 0.03^**^(0.01) | 0.03^**^(0.01) | 0.03^**^(0.01) |
| Frequency of social meetings (std.) |  | 0.06^***^(0.01) | 0.06^***^(0.01) | 0.06^***^(0.01) | 0.06^***^(0.01) | 0.06^***^(0.01) |
| Overall religiosity |  | -0.08^***^(0.01) | -0.08^***^(0.01) | -0.08^***^(0.01) | -0.08^***^(0.01) | -0.08^***^(0.01) |
| ***Country level*** |  |  |  |  |  |  |
| Conservation |  |  | 0.05 (0.03) |  |  |  |
| Openness to Change |  |  |  | -0.002 (0.03) |  |  |
| Self-Enhancement |  |  |  |  | -0.02 (0.03) |  |
| Self-Transcendence |  |  |  |  |  | 0.04 (0.03) |
| Constant | 0.16^*^(0.07) | 0.23^***^(0.07) | 0.22^***^(0.07) | 0.22^***^(0.07) | 0.22^***^(0.07) | 0.23^***^(0.07) |
|  | | | | | | |
| AIC | 108,160 | 97,308 | 97,245 | 97,246 | 97,245 | 97,244 |
| BIC | 108,220 | 97,479 | 97,500 | 97,501 | 97,501 | 97,500 |
| Variance of intercepts | 0.066 | 0.059 | 0.060 | 0.058 | 0.058 | 0.059 |
| Variance of Conservation |  |  | 0.003 | 0.003 | 0.003 | 0.003 |
| Variance of Self-Enhancement |  |  | 0.002 | 0.002 | 0.002 | 0.002 |
| Variance of female |  | 0.026 | 0.028 | 0.028 | 0.028 | 0.028 |
| Variance of age |  | 0.009 | 0.01 | 0.010 | 0.01 | 0.01 |
| Variance of residual | 0.884 | 0.760 | 0.758 | 0.758 | 0.758 | 0.758 |
|  | | | | | | |
| *Note.* ^*^p<0.05; ^**^p<0.01; ^***^p<0.001 |  | | | | | |

^a^ The models used non-ipsatized value scores at both levels.

# Table S10. Cross-level interactions between random effects and country-level four higher order values^a^

| **Individual-level effects** | **Country-level values** | | | |
| --- | --- | --- | --- | --- |
|  | Conservation | Openness to Change | Self-Enhancement | Self-Transcendence |
|  | | | | |
| Conservation | -0.02(0.01)* | -0.01(0.01) | -0.02(0.01)* | 0.00(0.01) |
| Openness to Change | 0.00(0.01) | -0.01(0.01) | -0.01(0.01) | 0.00(0.01) |
| Self-Enhancement | -0.02(0.01)** | 0.00(0.01) | -0.01(0.01) | 0.01(0.01) |
| Self-Transcendence | 0.00 (0.01) | -0.02(0.01)* | 0.00(0.01) | -0.01(0.01) |
| *Note.* ^*^p<0.05; ^**^p<0.01; ^***^p<0.001  ^a^ The models used non-ipsatized value scores at both levels. | | | |  |

# Figure S1. Frequency of alcohol consumption across 21 European countries


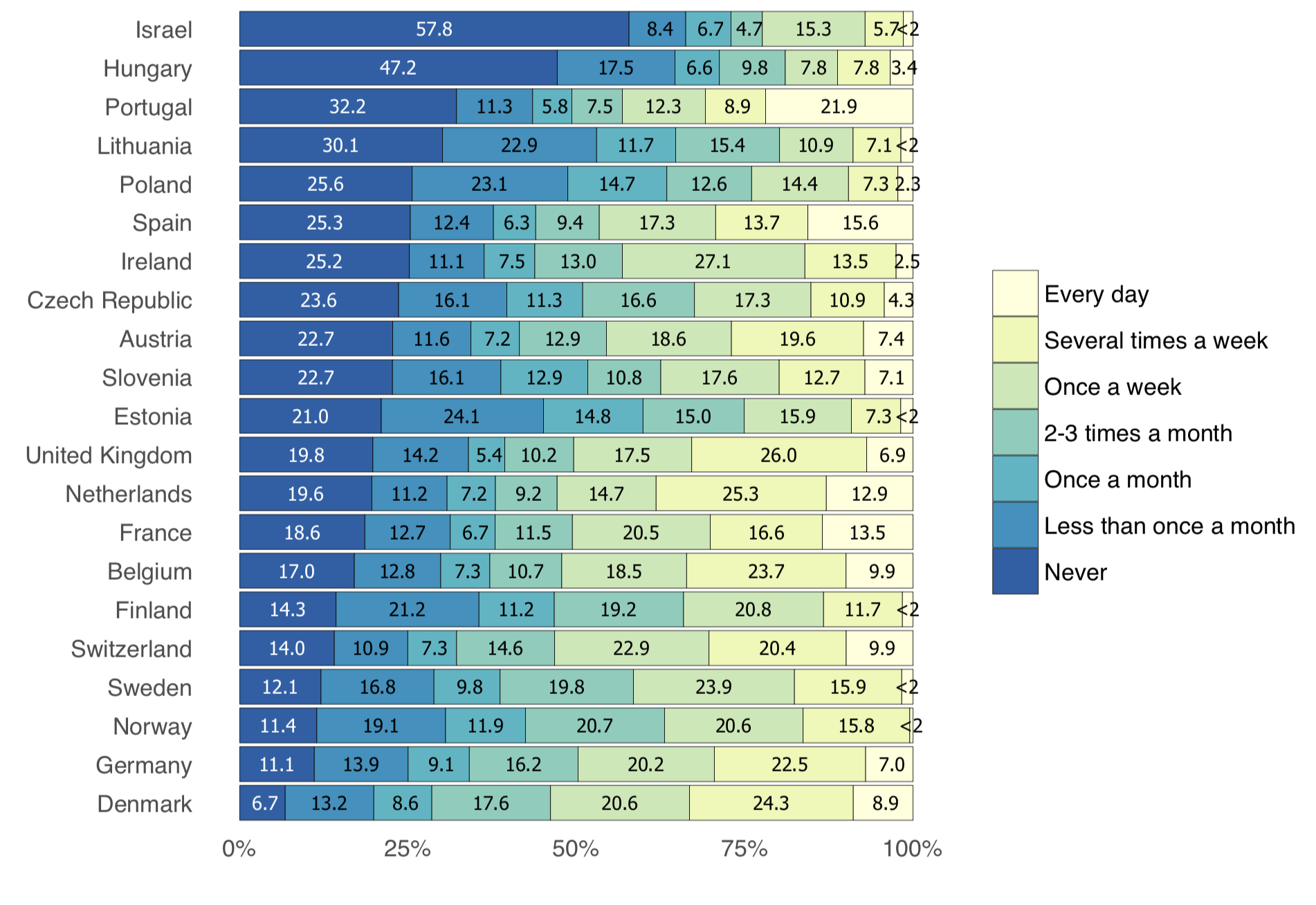

Supplement: Supplementary file 1 [file Table_1.docx]
